# Impacts of hypothyroidism on clinical, delivery, and fetal outcomes of coronavirus disease 2019: a cross-sectional study

**DOI:** 10.1590/1806-9282.20260677

**Published:** 2026-09-07

**Authors:** Suzan Şahin, Bülent Kaya, Emre Mat, Gazi Yıldız, Naile Gökkaya, Adil Barut

**Affiliations:** 1 Kartal Dr. Lütfi Kırdar City Hospital, Department of Infectious Diseases and Microbiology – Istanbul, Türkiye.; 2 Kartal Dr. Lütfi Kırdar City Hospital, Department of Obstetrics and Gynecology – Istanbul, Türkiye.; 3 Kartal Dr. Lütfi Kırdar City Hospital, Department of Endocrinology and Metabolism – Istanbul, Türkiye.; 4 Istanbul University-Cerrahpaşa, Cerrahpaşa Faculty of Medicine, Department of Obstetrics and Gynecology – Istanbul, Türkiye.

**Keywords:** COVID-19, Hypothyroidism, Pregnancy outcome, SARS-CoV-2

## Abstract

**OBJECTIVE:**

The aim of this study was to assess the effects of preexisting hypothyroidism on the course of coronavirus disease 2019 and on delivery and fetal outcomes in pregnant women with coronavirus disease 2019.

**METHODS:**

Two groups of pregnant women with coronavirus disease 2019 were included, that is, those with no comorbid condition and those with preexisting hypothyroidism as the sole comorbid condition. We assessed the course of coronavirus disease 2019 and delivery and fetal outcomes in the two groups.

**RESULTS:**

Of 167 participants, 143 had no preexisting comorbidity, and 24 had hypothyroidism as the sole comorbid condition. Patients with hypothyroidism had significantly higher incidences of severe coronavirus disease 2019 and the following coronavirus disease 2019 symptoms: shortness of breath, loss of sense of smell and/or taste, and tachypnea. Hospital (length of stay, intensive care unit admission and stay, mortality), delivery (normal vaginal delivery, cesarean section, and emergency cesarean section), and fetal (preterm, birth weight, 1- and 5-min Appearance, Pulse, Grimace, Activity, and Respiration scores, stillbirth) outcomes were similar in the two groups. The outcomes were also similar in patients with severe coronavirus disease 2019, though those with hypothyroidism had shorter hospital stay and intensive care unit stay and lower rates of intensive care unit admission, mortality, emergency cesarean section, preterm delivery, and low birth weight newborns.

**CONCLUSION:**

Our results suggest that preexisting hypothyroidism has adverse impacts on the severity of coronavirus disease 2019 and its symptoms. Considering severe coronavirus disease 2019 cases, seemingly favorable hospital, delivery, and fetal outcomes on the part of hypothyroidism may be worth further research.

## INTRODUCTION

During the coronavirus disease 2019 (COVID-19) pandemic, several studies investigated potential associations between COVID-19 and hypothyroidism in several aspects, including the risk imposed by hypothyroidism for contracting and for the prognosis of COVID-19. A comprehensive review from Denmark assessed relevant reports published during the first year of the pandemic and concluded that the presence of hypo- or hyperthyroidism was not associated with an increased risk of contracting COVID-19, nor with a worsened prognosis of COVID-19^1^. However, this has not been the case for the reverse relationship. There has been a consensus as to a pathogenetic link between COVID-19 and non-neoplastic thyroid diseases, including hypothyroidism, due to thyroid involvement^2,3,4^. Even the early reports from relatively small numbers of COVID-19 patients consistently found significantly lower levels of thyroid-stimulating hormone (TSH) and total triiodothyronine (TT3) in COVID-19 patients, closely correlated with the disease severity^5,6^.

As to pregnancy, delivery, and fetal outcomes of patients with COVID-19^7,8,9,10^ or with hypothyroidism^11,12^, adverse outcomes have been reported to be associated with COVID-19 or hypothyroidism, including higher rates of preterm deliveries, emergency cesarean section (CS), low birth weight newborns (14.5%) and stillbirths.

In our previous study of pregnant COVID-19 patients, preexisting hypothyroidism showed a remarkable coexistence not only with COVID-19 severity but also with adverse delivery outcomes, raising suspicion for a potential role in aggravating COVID-19 prognosis and pregnancy outcomes^13^. In a review of relevant literature, the authors found only a single study on the adverse effects of hypothyroidism in pregnant women with COVID-19^14^.

In the current study, the authors assessed whether preexisting hypothyroidism as the sole comorbid condition in pregnant women with COVID-19 adversely affected (i) the symptoms and the severity of COVID-19, and (ii) delivery and fetal outcomes as compared with patients with COVID-19 with no comorbid condition.

## METHODS

### Study design

We collected hospital data of two groups of patients who were admitted to the Obstetrics and Gynecology Clinic and had deliveries at our center between March 20, 2020, and January 1, 2023: (i) all pregnant women with COVID-19 with no preexisting comorbidity, and (ii) pregnant women with COVID-19 accompanied by preexisting hypothyroidism as the sole comorbid condition. Patients with comorbidities other than preexisting hypothyroidism were excluded. Comorbidities acquired during pregnancy (e.g., gestational hypothyroidism, gestational hypertension, gestational diabetes, and preeclampsia) were also excluded. The diagnosis of COVID-19 was confirmed by a Polymerase chain reaction (PCR) test in all the patients. The presence of preexisting hypothyroidism was defined as previous diagnoses of hypothyroidism made before conception of the participants.

Data included sociodemographic characteristics, number of pregnancies, type of pregnancy, gestational week, the presence or absence of preexisting hypothyroidism as the sole comorbid condition, the severity of COVID-19, vaccination status for COVID-19, the need for intensive care unit (ICU) admission, type and time of delivery, the need for emergency CS, mortality, 1- and 5-min Appearance, Pulse, Grimace, Activity, and Respiration (APGAR) scores, birth weight, and stillbirths. Anthropometric data of the patients were not recorded at the time of admission, and newborns were not tested for COVID-19.

### Definitions

The severity of COVID-19 was classified as asymptomatic, mild, moderate, or severe^7^. Patients with no COVID-19 symptoms were classified as asymptomatic. Flu-like symptoms (body temperature ≥38.3°C, cough, fatigue, headache and/or myalgias) and/or anosmia/ageusia were considered to show mild disease. Addition of dyspnea and decreased oxygenation (94 to <97%) suggested moderate disease. Decreased oxygenation below 94%, in addition to clinical symptoms and/or radiologic involvement of COVID-19, indicated severe disease. Radiological imaging was performed, avoiding radiation to the stomach only in patients who gave informed consent to radiologic assessment.

Delivery outcomes were abortus (<20 gestational weeks), preterm (20–36 weeks), and term (≥37 weeks) deliveries.

Low birth weight was defined as a birth weight of less than 2,500 g. Loss of a baby at or after 20 weeks of pregnancy was regarded as stillbirth.

### Ethical approval

The study was approved by the institutional review board (27.05.2024-2024/010/99/4/5), with exemption from obtaining patient consent because of its retrospective design and complete anonymity and de-identification of the participants. All the procedures concerning the study were conducted in compliance with the Declaration of Helsinki. Analysis and reporting of the results are in compliance with the Strengthening the Reporting of Observational Studies in Epidemiology (STROBE) checklist.

### Statistical analysis

Data were collected using a structured format, including relevant sociodemographic and clinical characteristics related to COVID-19 and hypothyroidism, and were analyzed using the Statistical Package for Social Sciences (SPSS) version 31.0 (IBM Corp., Armonk, NY, USA). Quantitative data were expressed as mean, standard deviation (SD), median, minimum, and maximum values, while qualitative data were presented as frequencies and percentages. The normality of continuous variables was assessed using the Shapiro-Wilk test. Homogeneity of variances was evaluated with Levene’s test, with a p>0.05 considered indicative of homogeneity. For comparisons between two groups, the independent samples t-test was used for normally distributed variables, whereas the Mann-Whitney U-test was applied for non-normally distributed variables. Categorical variables were analyzed using the Pearson’s chi-squared test. A p-value of <0.05 was considered statistically significant.

## RESULTS

During the study period, a total of 4,775 pregnant women were admitted to the Obstetrics and Gynecology Clinic. Among those, 295 had COVID-19, 193 of whom had deliveries at our center. Twenty-five patients with at least one comorbid condition other than preexisting hypothyroidism were excluded, including those with asthma (n=8), hypertension (n=7), diabetes mellitus (n=7), epilepsy (n=4), and bipolar disorder (n=1). One patient with preexisting hypothyroidism along with diabetes mellitus was also excluded. Thus, 167 patients met the inclusion criteria, that is, 143 COVID-19 patients with no preexisting comorbidity (Group 1) and 24 COVID-19 patients with hypothyroidism as the sole comorbid condition (Group 2).

### The incidence of hypothyroidism

The overall incidence of hypothyroidism among all pregnant patients with COVID-19 was 13.6% (40/295), with the majority of patients having hypothyroidism as the sole comorbid condition (11.9%, 35/295). The overall incidence of hypothyroidism among patients with COVID-19 who had deliveries at our center was 13.0 (25/193). After exclusion of patients with preexisting comorbidities other than hypothyroidism, the incidence was 14.4% (24/167). All patients with hypothyroidism had been on thyroid hormone therapy with levothyroxine both before and during pregnancy.

Characteristics of the two patient groups are summarized in Table 1. The two groups were similar with respect to age, vaccination rate for COVID-19, type of pregnancy, and gestational trimester. Comparison of the two groups showed similar findings with respect to abnormal laboratory parameters, hospital stay, ICU admission and ICU stay, and mortality.

**Table 1:** Patients’ characteristics (n=167).

Characteristics	COVID-19* (n=143)	COVID-19 plus hypothyroidism** (n=24)	p*
Age	28.9±5.4	30.9±4.9	0.103
Maternal characteristics			
Type of pregnancy, n (%)			
Primigravida	29 (20.3)	4 (16.7)	0.681
Multigravida	114 (79.7)	20 (83.3)	
Gestational trimester, n (%)			
First (1–13 weeks)	0	0	0.870
Second (14–26 weeks)	5 (3.5)	1 (4.2)	
Third (≥27 weeks)	138 (96.5)	23 (95.8)	
COVID-19 vaccinated	17 (11.9)	1 (4.2)	0.259
COVID-19 severity, n (%)			
Symptomless	86 (60.1)	13 (54.2)	**0.044**
Mild	49 (34.3)	6 (25)	
Moderate	1 (0.7)	0	
Severe	7 (4.9)	5 (20.8)	
COVID-19 symptoms, n (%)			
Cough	45 (31.5)	9 (37.5)	0.559
Fever	11 (7.7)	3 (12.5)	0.432
Shortness of breath	7 (4.9)	4 (16.7)	**0.031**
Headache	15 (10.5)	3 (12.5)	0.769
Myalgia	33 (23.1)	7 (29.2)	0.518
Diarrhea	0	0	-
Malaise	49 (34.3)	9 (37.5)	0.758
Loss of sense of smell/taste	2 (1.4)	2 (8.3)	**0.041**
Tachypnea	9 (6.3)	5 (20.8)	**0.017**
Abnormal laboratory parameters, n (%)			
Lymphopenia	35 (24.5)	8 (33.3)	0.358
C-reactive protein (>5 mg/L)	112 (78.3)	18 (75.0)	0.439
Lactate dehydrogenase (>214 U/L)	112 (78.3)	21 (87.5)	0.195
D-dimer (>550 μg/L)	134 (93.7)	24 (100)	0.464
Fibrinogen (>400 mg/dL)	82 (57.3)	15 (62.5)	0.929
Ferritin (>150 μg/L)	15 (10.5)	3 (12.5)	0.768
Hospital stay, days, median (IQR)	3.5 (3–5)	3 (3–4)	0.648
ICU admission, n (%)	6 (4.2)	2 (8.3)	0.380
ICU stay, days, median (IQR)	7.5 (3.5–17.5)	9 (4–9)	0.857
Mortality, n (%)	3 (2.1)	2 (8.3)	0.097
Type of delivery, n (%)			
Normal vaginal delivery	58 (40.6)	6 (25.0)	0.165
Cesarean section	85 (59.4)	18 (75.0)	
Emergency cesarean section	45 (31.5)	11 (45.8)	0.497
Delivery outcomes			
Delivery week (mean±SD)	36.6±4.0	36.8±4.5	0.747
Preterm, n (%)	31 (21.7)	6 (25.0)	0.418
Term, n (%)	108 (75.5)	18 (75.0)	
Fetal outcomes			
Stillbirth, n (%)	4 (2.8)	1 (4.2)	0.716
Birth weight (g)	2.994±710	3.080±606	0.598
Low birth weight, n (%)	26 (18.2)	3 (3.7)	0.662
APGAR 1 min (mean±SD)	7.5±1.6	7.5±1.6	0.985
APGAR 5 min (mean±SD)	8.7±1.5	8.6±1.8	0.881

*Patients with COVID-19 with no comorbid condition;

**Patients with COVID-19 with no other comorbid condition other than hypothyroidism. ICU, intensive care unit; COVID-19: coronavirus disease 2019; IQR: interquartile range; SD: standard deviation; APGAR: Appearance, Pulse, Grimace, Activity, and Respiration. *Figures in bold show a significant difference between the two groups.

### Coronavirus disease 2019 symptoms and severity

Concerning the severity and symptoms of COVID-19, there were significantly more patients with severe COVID-19 (20.8 vs. 4.9%, p=0.044) in Group 2 than in Group 1 as well as significantly higher incidences of the following COVID-19 symptoms: shortness of breath (16.7 vs. 4.9%, p=0.031), loss of sense of smell and/or taste (8.3 vs. 1.4%, p=0.041), and tachypnea (20.8 vs. 6.3%, p=0.017).

### Delivery outcomes

Patients in Group 1 and Group 2 had similar delivery weeks and similar rates of preterm and term deliveries. Patients with preexisting hypothyroidism had a lower rate of normal vaginal delivery (25.0 vs. 40.6%) and higher rates of CS (75.0 vs. 59.4%) and emergency CS (45.8 vs. 31.5%), but none reached a significant difference.

### Fetal outcomes

Among fetal outcomes, the two groups were similar with respect to the values of birth weight and 1- and 5-min APGAR scores. Group 1 exhibited a higher rate of low birth weight newborns (18.2 vs. 3.7%), whereas Group 2 had a higher rate of stillbirth (4.2 vs. 2.8%), but none yielded a significant difference.

### Comparison of patients with severe coronavirus disease 2019

Table 2 presents hospital data and fetal outcomes of two patient groups with severe COVID-19 with or without hypothyroidism with no other comorbid condition. Although almost all parameters assessed were more favorable in patients with severe COVID-19 and hypothyroidism, none reached statistical significance. Patients with severe COVID-19 who did not have hypothyroidism had a longer hospital stay (mean 16.6 vs. 10.6 days) and ICU stay (mean 9.6 vs. 9 days), as well as higher rates of ICU admission (85.7 vs. 40.0%), mortality (42.9 vs. 40%), emergency CS (85.7 vs. 40.0%), preterm deliveries (71.4 vs. 20.0%), and low birth weight newborns (71.4 vs. 0%). These patients also had a lower rate of term deliveries (28.6 vs. 60.0%) as well as lower newborn mean APGAR 1-min (4.3 vs. 7.5) and 5-min (6.5 vs. 9.0) scores. The only more adverse outcome seen in those with hypothyroidism was the incidence of stillbirth, which occurred in one patient (20.0%) versus no patient.

**Table 2:** Comparison of patients with severe coronavirus disease 2019 (n=12).

Characteristics	Severe COVID-19* (n=7)	Severe COVID-19 plus hypothyroidism** (n=5)	p
Hospital stay, days, median (range)	16.0 (6–31)	9.0 (5–24)	0.202
ICU admission, n (%)	6 (85.7)	2 (40.0)	0.098
ICU stay, days, median (range)	7.5 (2–19)	9.0 (4–14)	0.857
Mortality, n (%)	3 (42.9)	2 (40.0)	0.921
Delivery outcomes			
Emergency cesarean section, n (%)	6 (85.7)	2 (40.0)	0.098
Fetal outcomes, n (%)			
Preterm	5 (71.4)	1 (20.0)	0.276
Term	2 (28.6)	3 (60.0)	0.276
Stillbirth	0	1 (20.0)	0.216
Low birth weight	5 (71.4)	0	0.022
APGAR 1 min, median (range)	4.0 (1–8)	7.0 (0–8)	0.662
APGAR 5 min, median (range)	6.5 (3–9)	9.0 (0–9)	0.429

*Patients with severe COVID-19 with no comorbid condition;

**Patients with severe COVID-19 with no other comorbid condition other than hypothyroidism. ICU, intensive care unit; COVID-19: coronavirus disease 2019; APGAR: Appearance, Pulse, Grimace, Activity, and Respiration.

## DISCUSSION

The present study was designed upon intriguing detection, in our previous study^13^, of coexistence of hypothyroidism with COVID-19 in pregnant women in terms of severity of COVID-19 and its symptoms and obstetric and neonatal outcomes. The authors noted that (i) the incidence of hypothyroidism was considerably higher among patients with moderate-severe COVID-19; (ii) none of the patients requiring ICU admission had any comorbidity other than hypothyroidism; (iii) nearly half of mortality cases due to severe COVID-19 occurred in patients who had hypothyroidism, but no other comorbidity; (iv) hypothyroidism was the most frequent comorbid condition among patients who required emergency CS; (v) patients with hypothyroidism had strikingly high incidences of preterm delivery, low-birth-weight newborns, and stillbirth^13^. Based on these findings in pregnant women with COVID-19, the authors hypothesized that preexisting hypothyroidism might have an additive adverse effect on the course and severity of COVID-19 as well as on delivery and fetal outcomes.

In our study, the incidence of severe COVID-19 was significantly higher among those with hypothyroidism (20.8 vs. 4.9%), as were the incidences of COVID-19 symptoms (shortness of breath 16.7 vs. 4.9%); loss of sense of smell and/or taste (8.3 vs. 1.4%; and tachypnea 20.8 vs. 6.3%). On the other hand, the presence of hypothyroidism was associated with a longer ICU stay and higher rates of ICU admission, mortality, CS and emergency CS, preterm deliveries, and stillbirth, but these did not reach statistical significance (Table 1).

Interestingly, when only patients with severe COVID-19 are taken into consideration, those without hypothyroidism had more adverse outcomes characterized by longer hospital and ICU stay, higher rates of ICU admission, mortality, emergency CS, preterm delivery, and low birth weight newborns, as well as lower 1-min APGAR scores (Table 2).

To date, the potential role of hypothyroidism in the setting of COVID-19 and pregnancy has been assessed in a single study, which included 145 COVID-19-positive pregnant women (vs. 167 in the present study), 13 of whom (vs. 24 in the present study) also had hypothyroidism^14^. Although the authors reported an incidence of 8.96% in their study, a careful examination revealed that 2 of 13 patients with hypothyroidism reported also had gestational diabetes mellitus, reducing the incidence of hypothyroidism as the sole comorbid condition to 7.6%, as compared with 14.4% in our study. The authors found no significant differences between COVID-19 patients with and without hypothyroidism in terms of delivery and fetal outcomes (birth weight, 1-min APGAR score). Pneumonia and ICU admission were seen only in COVID-19 patients without hypothyroidism, who also had longer hospitalization. Interestingly, the finding that seemingly more adverse outcomes were seen in patients without hypothyroidism in the setting of COVID-19 is in line with our findings in severe COVID-19 patients without hypothyroidism, who also had seemingly more adverse outcomes in many aspects, including hospital stay, ICU admission, mortality, emergency CS, preterm delivery, low birth weight, and APGAR 1- and 5-min scores. Concerning stillbirth, similar to our finding, the authors reported one case in COVID-19 patients with hypothyroidism. One striking difference is in the incidence of emergency CS in the setting of COVID-19, in that, in the abovementioned study, patients with hypothyroidism had a higher incidence of emergency CS in contrast with our finding of a more than two-fold higher incidence in patients without hypothyroidism.

### Limitations

The main limitations of the present study are its retrospective design and the small number of patients with COVID-19 and hypothyroidism. Yet, the small patient size does not necessarily preclude attempts at research in certain conditions. Although hypothyroidism has been reported as one of the most frequent comorbid conditions among pregnant patients with COVID-19, evidence is scarce about its potential role in the severity of COVID-19 and in delivery and fetal outcomes. The only previous study assessing hypothyroidism in the setting of COVID-19 with delivery and fetal outcomes had a patient size of 13 and a percentage of 8.96%, as compared with our patient size of 24, accounting for 14.4%.

In conclusion, taking into consideration relevant literature reports, the adverse effects of hypothyroidism on pregnant women with COVID-19 may be understandable, but this may not be the case in the setting of severe COVID-19, where hypothyroidism may have seemingly favorable effects on clinical, delivery, and fetal outcomes. Our findings suggest that the mechanisms, if any, of seemingly “protective” effects of hypothyroidism in pregnant women with severe COVID-19 on clinical, delivery, and fetal outcomes may be worth further investigation and studies.

## Data Availability

The datasets generated and/or analyzed during the current study are available from the corresponding author upon reasonable request.
